# Drug resistance from preferred antiretroviral regimens for HIV infection in South Africa: A modeling study

**DOI:** 10.1371/journal.pone.0218649

**Published:** 2019-07-03

**Authors:** Ume L. Abbas, Robert L. Glaubius, Yajun Ding, Gregory Hood

**Affiliations:** 1 Department of Medicine, Section of Infectious Diseases and Department of Molecular Virology and Microbiology, Baylor College of Medicine, Houston, Texas, United States of America; 2 Departments of Quantitative Health Sciences and Infectious Disease, Cleveland Clinic, Cleveland, Ohio, United States of America; 3 Pittsburgh Supercomputing Center, Carnegie Mellon University, Pittsburgh, Pennsylvania, United States of America; University of KwaZulu-Natal, SOUTH AFRICA

## Abstract

**Background:**

Tenofovir-containing regimens comprise the preferred first-line antiretroviral therapy (ART) in many countries including South Africa, where utilization of second-line regimens is limited. Considerable HIV drug resistance has occurred among persons failing tenofovir-containing first-line ART. We evaluated drug resistance at the population level using mathematical modeling.

**Setting:**

Heterosexual HIV epidemic in KwaZulu-Natal, South Africa.

**Methods:**

We constructed a stochastic individual-based model and simulated scenarios of ART implementation, either CD4-based (threshold < 500 cells/mL) or Fast-track (81% coverage by 2020), with consideration of major drug-associated mutations (M184V, K65R and non-nucleoside reverse transcriptase inhibitor (NNRTI)). Using base case and uncertainty analyses, we assessed (majority) drug resistance levels.

**Results:**

By 2030, the median total resistance (proportion of HIV-infected persons with drug resistance) is predicted to reach 31.4% (interquartile range (IQR): 16.5%-50.2%) with CD4-based ART, decreasing to 14.5% (IQR: 7.7%-25.8%) with Fast-track implementation. In both scenarios, we find comparably high prevalence (~80%) of acquired NNRTI-associated, M184V and K65R mutations. Over 48% of individuals with acquired resistance harbor dual, 44% triple and 7% just single drug mutations. Drug-resistant HIV is predicted to comprise 40% (IQR: 27%-50%) of incident infections, while 70% of prevalent transmitted resistance is NNRTI-associated. At 2018, the projected total resistance is 15% (IQR: 7.5%-25%), with 18% (IQR: 13%-24%) of incident infections from transmitted drug-resistant HIV.

**Conclusions:**

WHO-recommended preferred first-line ART could lead to substantial drug resistance. Effective surveillance of HIV drug resistance and utilization of second-line as well as alternative first-line regimens is crucial.

## Introduction

Global HIV incidence, though slowly receding, remains unacceptably high at 1.8 million new infections annually [1]. The remarkable efficacy of antiretroviral therapy (ART) for both HIV treatment and prevention led the Joint United Nations Programme on HIV/AIDS (UNAIDS) to recommend a Fast-track approach to ending the AIDS epidemic as a public health threat by 2030, and to establish ambitious targets including 90-90-90 by 2020 (90% of HIV-positive people know their serostatus, of whom 90% receive sustained ART, and 90% of these have viral suppression) and 95-95-95 by 2030 [2]. However, the expansion and maturity of ART programs globally are associated with a rise in HIV drug resistance, posing a threat to the success of ART scale-up and the overall HIV response [3]. Therefore, the World Health Organization (WHO) has launched a Global Action Plan [4], providing a framework, interventions and resources for counteraction, including the recommended consideration of change in the preferred ART regimens [5], especially in countries where the prevalence of pretreatment HIV drug resistance exceeds 10% (detected in antiretroviral naïve or antiretroviral exposed individuals initiating or reinitiating first-line ART) [6]. Concerns about HIV drug resistance are particularly relevant to the large epidemic in South Africa, where only 61% of the HIV-positive people were on treatment in 2017 [1], and in its hardest-hit province of KwaZulu-Natal, where 28% of adults are HIV-positive [7]. In this region, the scale, pace and nature of drug resistance at the population-level are unclear, amidst disparate data [8–17]. Therefore, we employed mathematical modelling to study HIV drug resistance from ART implementation in KwaZulu-Natal, South Africa.

## Methods

We constructed and analyzed a stochastic individual-based mathematical model of the HIV epidemic in KwaZulu-Natal, with details of ART scale-up and HIV drug resistance, using discrete event systems modeling and simulation [18–26]. This stochastic model is founded as an analogue [27–30] to our prototype deterministic model of the HIV epidemic in KwaZulu-Natal [31, 32], and is detailed to extend and refine our modeling of antiretroviral drug resistance [31–33]. Below, we describe the stochastic model structure, assumptions and analytic design, pertinent to this study. Complete specification of our deterministic HIV epidemic model of KwaZulu-Natal is available elsewhere [31–33].

### Model structure

**Fig 1:**
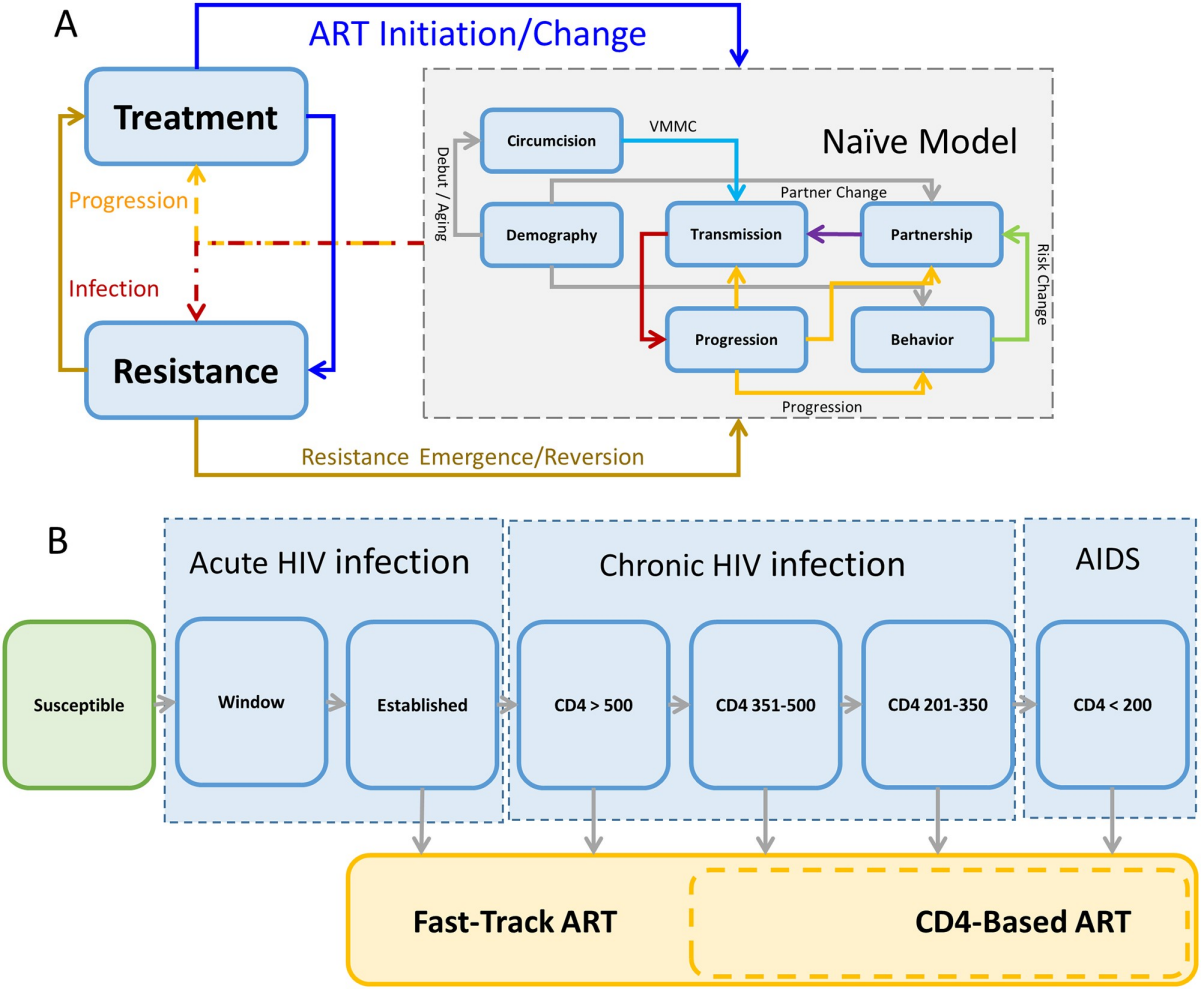
Model structure. A: Modular structure of the discrete event simulation model. B: Simplified model flow diagram.

**Fig 2:**
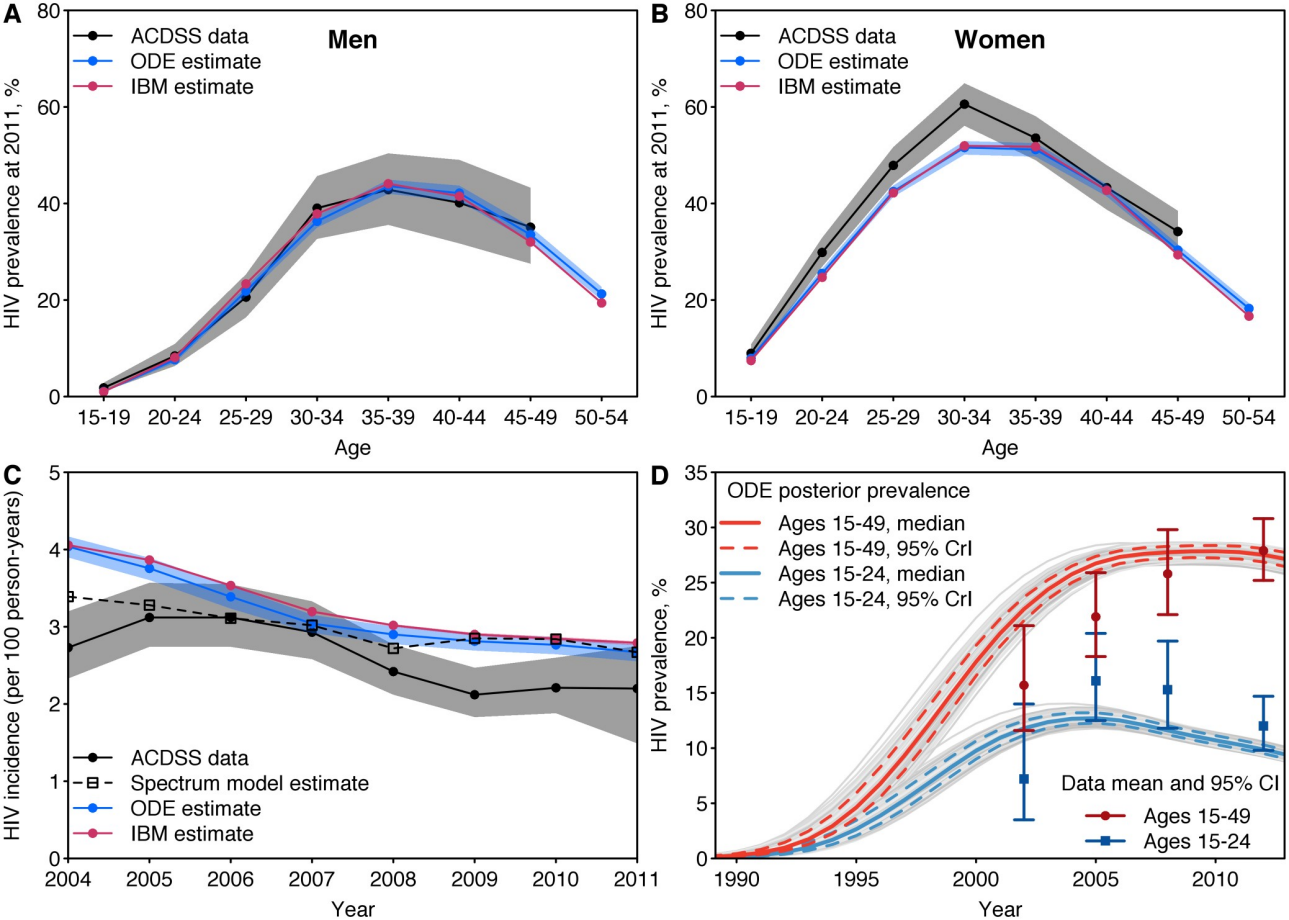
Calibration of the deterministic model and the stochastic individual-based model. Model calibration to HIV prevalence among (A) women and (B) men by age. Error bars show 95% confidence intervals for data and 95% credible intervals for the IBM model (imperceptible because narrow) and ODE model (posterior) estimates. C: Model calibration to HIV incidence in the ACDSS and comparison to the UNAIDS’ Spectrum model [40]. D: Model validation against HIV prevalence in KwaZulu-Natal among adults aged 15–24 and 15–49 from the four South African national household surveys [7]. Abbreviations: ODE, ordinary differential equation; IBM, individual based model; ACDSS, Africa Centre Demographic Surveillance Site; CrI, credible interval.

The model is comprised of a set of modules that represent different categories of dynamic processes such as demographics, sexual behavior change, HIV transmission, disease progression, drug resistance and interventions for HIV prevention and treatment (Fig 1, Table 1 and S1 Table). The model tracks over time, the life histories of all individuals in a realistically sized population (2.5 million initially), characterized by various features (attributes) including gender, age (15–54 years), sexual behavior, infection status, disease stage, intervention status including first- and second-line ART, voluntary medical male circumcision (VMMC) and HIV drug susceptibility. HIV transmission is represented through heterosexual contact influenced by mixing patterns and behavioral factors including condom-use. The overall model state is updated dynamically based on randomly occurring events having specified rates [34] and about exponentially distributed time to next event [35, 36]. The stochastic model is specified using discrete event system specification (DEVS) [20–22] and C++ [37]. It is formulated and parameterized as a stochastic analogue [27–30] to our prototype deterministic model [31, 32] that was calibrated earlier using Bayesian methods [38]; thereby using the deterministic model’s median posterior input estimates [38] and fitting [39] to simulate the longitudinal HIV incidence and prevalence in KwaZulu-Natal and cross-sectional behavioral risk stratified HIV prevalence in South Africa (Fig 2).

**Table 1:** Key intervention-related model parameters.

Input	Base Case	Uncertainty Range: LHS	Reference
**VMMC**			
Male circumcision prevalence at Jan. 1, 2021, %	80	60–85	[7, 41]
VMMC effectiveness against male HIV acquisition, %	60	–	[42]
			
**ART**			
ART coverage of CD4 ≤ 500 cells/μL at Jan. 1, 2021, %	80	65–80	[41, 43]
Time Fast-track implementation begins, year	Sep 1, 2016	–	[44]
Fast-track ART uptake rate, % per year	50	–	[45]
Decrease in Fast-track ART virologic failure due to adherence support, %	80	–	[45]
Fast-track ART coverage of PLHIV at 2021, %	81	–	[46]
Fast-track ART coverage of PLHIV at 2031, %	90	–	[46]
ART effectiveness against HIV transmission while suppressed, %	96	73–99	[47]
Dropout rate during the first year of 1^st^-line ART, per year	0.15	0.1–0.2	[48]
Dropout rate during subsequent years of 1^st^-line ART, per year	0.08	0.04–0.12	[48]
Dropout rate while non-adherent to 1^st^-line ART, per year	0.08	0.04–0.12	[48]
HIV mortality rate during the first year of 1^st^-line ART if ART initiated at CD4 ≤ 200 cells/μL, per year	0.15	0.1–0.2	[49]
HIV mortality rate during subsequent years of 1^st^-line ART if ART initiated at CD4 ≤ 200 cells/μL, per year	0.03	0.02–0.06	[49]
HIV mortality rate on ART if ART initiated at 201–350 CD4 cells/μL, relative to ART initiated at CD4 ≤ 200 cells/μL, %	33	15–85	[50]
HIV mortality rate on ART if ART initiated at 351–500 CD4 cells/μL, relative to ART initiated at 201–350 CD4 cells/μL, %	88	62.5–100	[51]
Virologic failure rate during the first year of 1^st^-line ART while harboring wild-type HIV, % per year	20	10–30	[52]
Virologic failure rate during subsequent years of 1^st^-line ART while harboring wild-type HIV, % per year	5	2.5–7.5	[53]
Virologic failure rate during the first year of 1^st^-line ART while harboring drug-resistant HIV, % per year	40	15–75	[54, 55]
Virologic failure rate during subsequent years of 1^st^-line ART while harboring drug-resistant HIV, % per year	10	3.75–22.5	[54]
Proportion of 1^st^-line ART virologic failure that is due to non-adherence, %	28	**10–50**	[52, 56–58]
			
**HIV Drug Resistance**			
**Persistence time of transmitted NNRTI resistance, years**	18	**5–56**	[59]
**Persistence time of acquired NNRTI resistance, years**	1	**0.5–5.0**	[60–62]
**Persistence time of transmitted M184V resistance, years**	1	**0.5–2**	[63]
**Persistence time of acquired M184V resistance, years**	0.25	**0.125–0.375**	[60, 64, 65]
**Persistence time of transmitted K65R resistance, years**	1.43	**0.71–2.86**	Calculated
**Persistence time of acquired K65R resistance, years**	0.36	**0.18–0.54**	[65]
**Fitness cost of transmitted drug resistance, % reduction**	0	-	[66]
**Fitness cost of acquired NNRTI resistance, % reduction**	25	**25–70**	[60, 67]
**Fitness cost of acquired M184Vresistance, % reduction**	65	**60–70**	[60, 67]
**Fitness cost of acquired K65R resistance, % reduction**	50	**45–60**	[68]
**Disease progression rate with HIV having transmitted resistance to 1^st^-line ART, relative to wild-type HIV, %**	100	**50–100**	Assumed
**Disease progression rate with HIV having acquired resistance to 1^st^-line ART, relative to wild-type HIV, %**	Variable	**Variable**	Per fitness cost
**Resistance spectrum after first-line treatment failure (wild-type, K65R, M184V, NNRT, K65R-M184V, K65R-NNRTI, M184V-NNRTI, K65R-M184V-NNRTI), %**	28, 2.3, 0.5, 0.5, 10.2, 10.7, 15, 32.8	**Variable**	[69]

ART, antiretroviral therapy; LHS, Latin hypercube sampling; VMMC, voluntary medical male circumcision. Parameter estimates varied during uncertainty analyses for the current study are shown in bold text.

### HIV drug resistance

The model distinguishes HIV-positive individuals by antiretroviral use (not on ART or on ART), HIV drug susceptibility (drug-sensitive or drug-resistant), type of drug resistance (transmitted or acquired), and virus population dynamics of drug-resistant HIV (majority or minority). Drug-resistant virus is either acquired through selection pressure from ART or transmitted from a donor with drug-resistant HIV. Drug-resistant virus can revert to drug-sensitive wild-type, off of ART or in a new host, but archived resistance can re-emerge with subsequent ART exposure. We assume that drug-resistant infection can reduce the efficacy of treatment (Table 1). The model represents the presence or absence of the major drug resistance mutations [70], either singly or in combination, associated with antiretrovirals in the WHO recommended preferred first and second-line ART regimens [71], excluding the alternative and/or interim recommended dolutegravir (DTG)-containing regimens [4, 5]. Thus, we consider tenofovir disoproxil fumarate (TDF) + lamivudine or emtricitabine (XTC) + efavirenz or nevirapine (EFV/NVP) as the first-line ART regimen, and zidovudine (AZT) + XTC + boosted lopinavir (LPV/r) as the second-line ART regimen. The following drug resistance mutations (associated with antiretrovirals) are modeled: i) Nucleoside reverse transcriptase (NRTI)-associated signature mutations [70]—M184V (XTC), K65R (TDF) and the thymidine analogue mutations / TAMs (AZT); ii) Non-nucleoside reverse transcriptase (NNRTI)-associated class mutations (EFV/NVP); and iii) Protease inhibitor (PI)-associated class mutations (LPV) [3]. Our assumptions regarding the emergence of acquired resistance to first-line ART are primarily informed by the TenoRes study [69] (Table 1), while those for resistance to second-line ART (not pertinent to this work) are informed by the SELECT study [72]. Though South Africa has the largest ART program in the world, less than 5% of HIV-positive individuals are on second-line regimens [73]. Thus, for clarity and focus on drug resistance from first-line ART, we do not implement the scale-up of second-line ART in this study.

### Model-based analyses

#### CD4-based ART scenario

Fifty-six percent of HIV-positive people were receiving ART in South Africa in 2016 [74], increasing to 61% in 2017 [1]. Thus, we assume a conservative ART scenario based on South Africa’s 2012–2016 National Strategic Plan [41], achieving 80% VMMC coverage among men and 80% ART coverage among HIV-positive individuals with CD4 cell counts ≤500 cells/μL [43] by 2020, with maintenance thereafter. We assume that VMMC reduces the risk of HIV acquisition in men by 60% [42] and that suppressive ART reduces the transmission risk by 96% [47] and prolongs the survival of HIV-positive individuals [49–51, 75, 76].

#### Fast-track ART scenario

In 2016, South Africa expanded treatment eligibility to include all HIV-positive individuals regardless of CD4 cell count, and adopted the UNAIDS Fast-track treatment targets [44]. Therefore, we simulate an accelerated ART scenario, assuming 80% VMMC implementation plus universal ART eligibility with expanded testing and treatment rollout, beginning in September 2016 and reaching Fast-track treatment coverage targets of 81% by 2020 and 90% by 2030. To achieve the corresponding Fast-track targets of 73% and 86% overall virologic suppression, we assume an aspirational concurrent adherence-support intervention that reduces virologic failure rates 50% by 2020 and by 80% ultimately (relative to conservative scenario (Table 1)) [77].

### Study outcomes

We simulated the CD4-based ART and Fast-track ART, using 100 independent replications [18, 19] for each scenario, from 1978 to 2030 (base case analyses). Next, we repeated the above replications, with random variation of select resistance-related parameters over a specified range (Table 1), using Latin Hypercube Sampling [78], to determine the extent of uncertainty in model results (uncertainty analyses). We calculated the model output (outcome variables) means for base case, and the medians with interquartile range (IQR) for uncertainty analyses. Again, while our model incorporates both first- and second-line ART, the focus of this study is drug resistance associated with preferred first-line ART, reflecting limited utilization of second-line and alternative first-line regimens in South Africa [74, 79, 80]. Our primary outcome is the prevalence of HIV drug resistance in the majority virus by the end of 2030. Additionally, we assess the incidence of drug resistance and the resistance levels at 2018.

#### Outcome variable definitions

Total resistance prevalence is defined as the number of HIV-positive individuals with virological non-suppression and acquired and/or transmitted drug-resistant majority virus, divided by the entire number of HIV-positive individuals, at a given time.

Prevalence of acquired resistance is specified as the number of HIV-positive individuals with virological non-suppression and acquired drug-resistant majority virus, divided by the entire number of HIV-positive individuals, at a given time.

Prevalence of acquired drug resistance mutations represents the number of HIV-positive individuals with virological non-suppression and majority virus harboring acquired NNRT-class, K65R and/or M184V mutations (occurring as single or multiple drug-resistant viral variants/mutants), divided by the number of HIV-positive individuals with virological non-suppression and acquired drug-resistant majority virus, at a given time.

Incidence of transmitted resistance is specified as the proportion of new infections due to drug-resistant HIV, in a given time. The prevalence of transmitted resistance is the number of HIV-positive individuals with virological non-suppression and transmitted drug-resistant majority virus, divided by the entire number of HIV-positive individuals, at a given time.

Prevalence of ART-adjusted (total, acquired or transmitted) drug resistance is defined as the number of HIV-positive individuals with virological non-suppression and (acquired and/or transmitted) drug-resistant majority virus, divided by the number of HIV-positive individuals with ART-experience and virological non-suppression, at a given time.

## Results

### Base case analyses

#### Prevalence of HIV drug resistance

**Fig 3:**
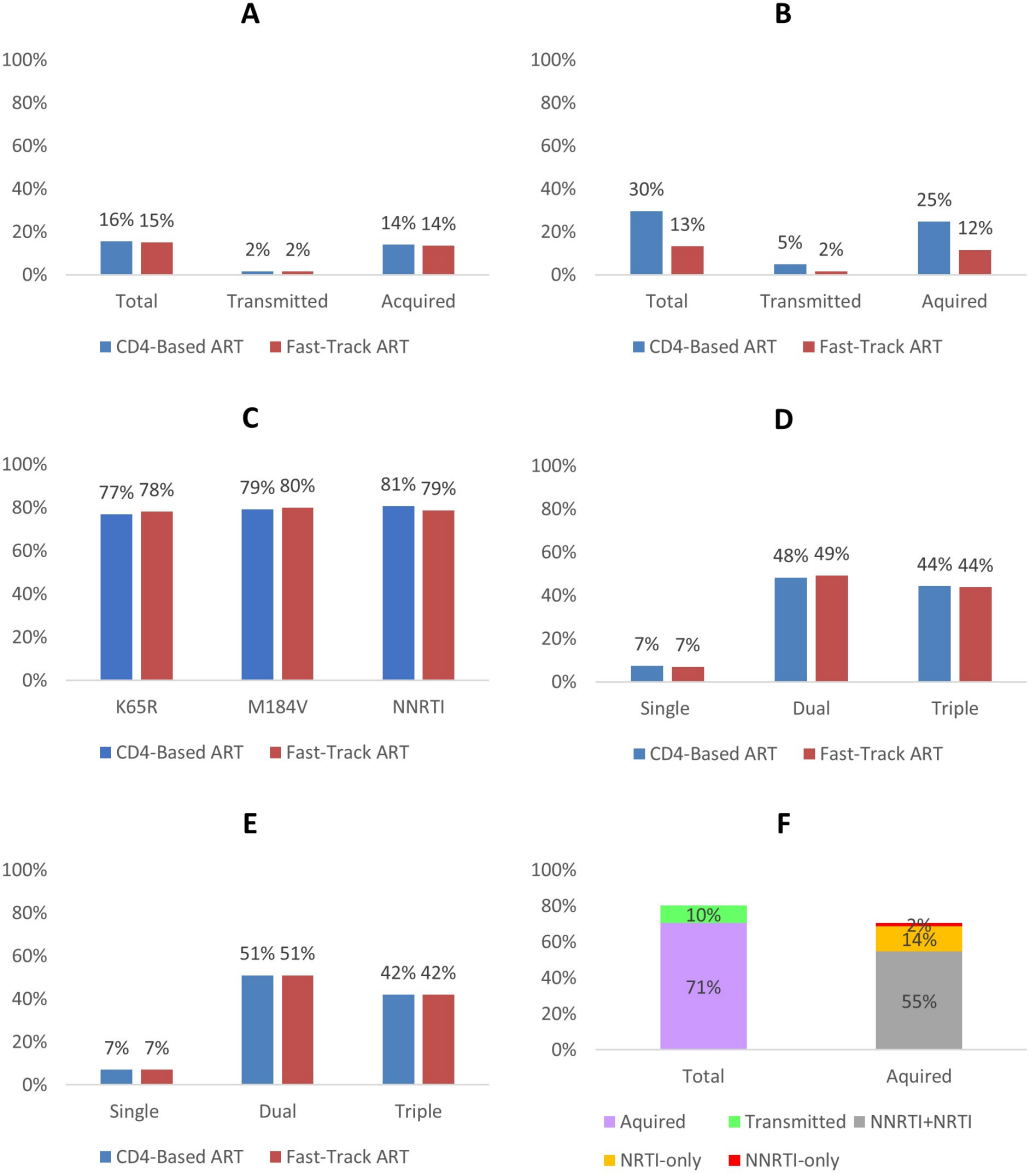
Prevalence of total, acquired and ART-adjusted HIV drug resistance. A: Prevalence of total drug resistance at 2018. B: Prevalence of total drug resistance by 2030. C: Prevalence of acquired drug resistance mutations by 2030. D: Prevalence of acquired drug-resistant mutants by 2030. E: Prevalence of acquired drug-resistant mutants at 2018. F: ART-adjusted drug resistance prevalence at 2018.

At 2018, the total resistance prevalence was similar in the two scenarios (~15%; ~230, 000 cases), with over a tenth contributed by transmitted resistance (~1.5%) (Fig 3A).

By 2030, the total resistance prevalence reached 30% from CD4-based ART (~375, 000 cases), while Fast-track ART reduced the total resistance to 13% (~150, 000 cases). In either scenario, over four-fifths of the total resistance was attributable to acquired resistance (Fig 3B).

#### Prevalence of acquired HIV drug resistance mutations

Irrespective of the scenario, the prevalence of the NNRTI-associated (class), M184V and K65R (signature) mutations was comparable (~80%) in individuals with acquired resistance and virological non-suppression, by 2030 (Fig 3C). Among these individuals, over 48% harbored dual drug mutations, 44% had triple mutations and 7% just single mutations (Fig 3D). Similarly, at 2018, the proportions of these individuals with dual, triple and single mutations were 51%, 42% and 7% respectively (Fig 3E).

#### Prevalence and incidence of transmitted HIV drug resistance

**Fig 4:**
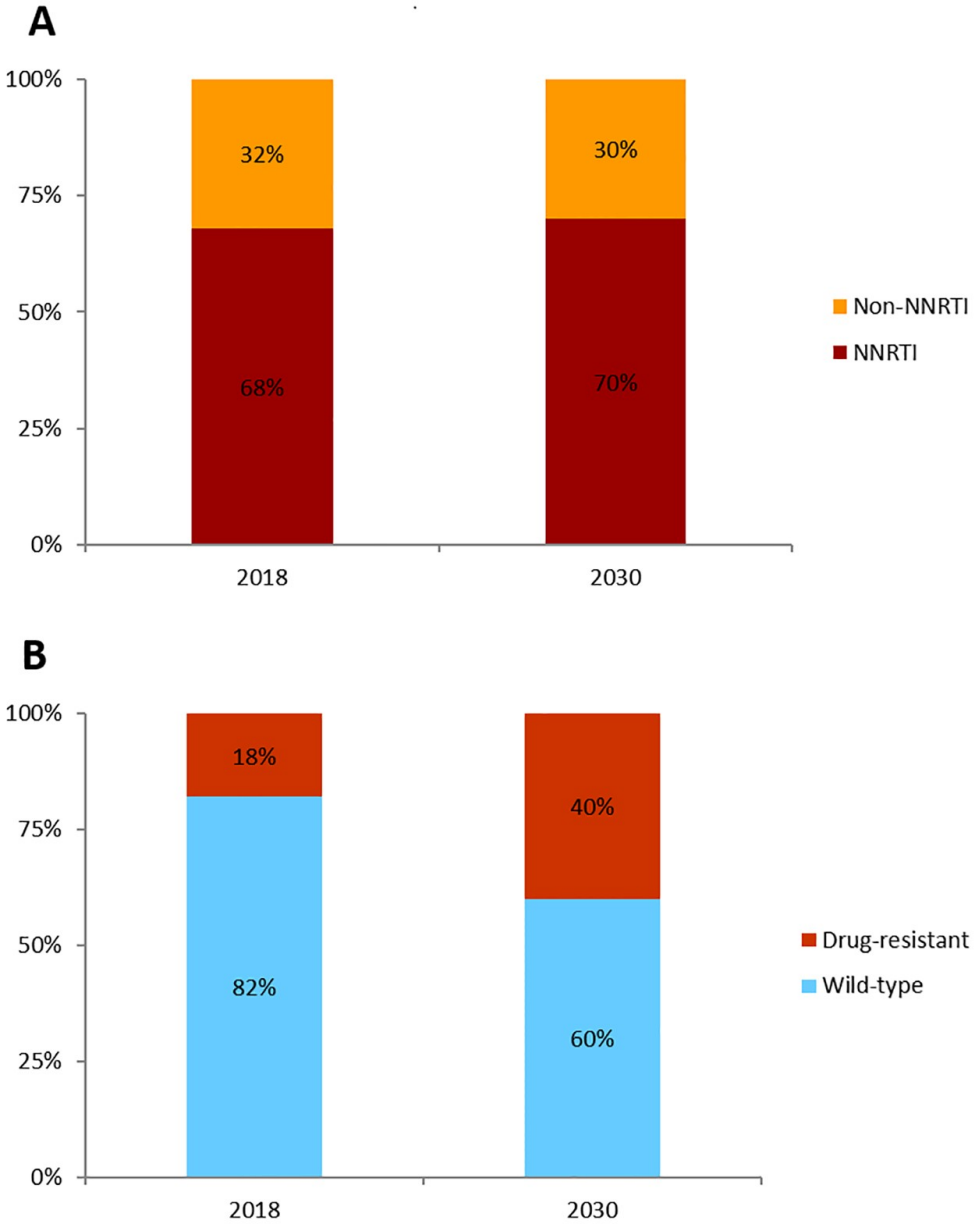
Prevalence and incidence of transmitted HIV drug resistance. A: Distribution of transmitted drug resistance by resistance type at 2018 and by 2030. B: Distribution of incident infections by resistance status at 2018 and by 2030.

Transmitted drug resistance was relatively similar in the two scenarios. NNRTI-associated (class) mutations comprised about 70% of the prevalent transmitted drug resistance at 2018 and end of 2030 (Fig 4A). At 2018, an estimated 18% of the incident/new infections (incidence) were due to transmitted drug-resistant HIV (Fig 4B). By the end of 2030, the proportion of incident drug-resistant HIV infections had risen to 40% (Fig 4B).

#### ART-adjusted HIV drug resistance

In both scenarios at 2018, the ART-adjusted total resistance prevalence was approximately 80%; comprising about 10% transmitted and 70% acquired resistance (Fig 3F). NNRTI-only (transmitted and acquired) resistance prevalence was about 8% (data not shown) while acquired NNRTI+NRTI resistance prevalence was about 55% (Fig 3F).

### Uncertainty analyses

The results of our base case analyses were confirmed by uncertainty analyses and are described below.

#### CD4-based ART scenario

At 2018, the median total resistance prevalence in this scenario was at a level of 15.5% (IQR: 7.5%-25.3%), constituted by 13.9% (6.6%-22.7%) acquired and 1.6% (0.9%-2.5%) transmitted resistance, and comprising 247, 577 (117, 820–410, 492) resistance cases. By 2030, the median total resistance reached 31.4% (IQR: 16.5%-50.2%). This comprised 399, 504 (205, 366–654, 732) resistance cases and constituted 27% (14.3%-43.2%) acquired and 4.5% (2.3%-7%) transmitted resistance. The median proportion of incident infections attributable to transmitted drug-resistant HIV was 18.3% (IQR: 13%-23.7%) at 2018 which increased to 38.1% (IQR: 26.9%-49.2%) by 2030.

#### Fast-track ART scenario

Similar to CD4-based ART, at 2018, the median total resistance prevalence in Fast-track scenario was 15.1% (IQR: 7.5%-25.3%), with an estimated 240,501 (114,615–399,151) cases and 13.5% (6.5%-22.1%) acquired and 1.5% (0.9%-2.5%) transmitted resistance. By 2030, the total resistance from Fast-track ART was less compared to 2018, and considerably less compared to the total resistance from CD4-based ART by 2030; its median value was 14.5% (IQR: 7.7%-25.8%), comprising 163,415 (84,784–299,174) cases of resistance and 13% (6.9%-23.6%) acquired and 1.5% (0.8%-2.2%) transmitted resistance. At 2018, the median proportion of drug-resistant incident infections was 17.9% (IQR: 12.9%-23.4%), increasing to 40.6% (IQR: 28.7%-49.5%) by 2030.

#### Survey-based versus model-based HIV drug resistance

S1 Fig illustrates data from the 5th South African National HIV Prevalence, Incidence, Behaviour and Communication Survey, 2017; a cross-sectional, population-based, household survey [81]. Among all survey participants who were HIV-positive with virological non-suppression and successful drug resistance testing, the weighted total (any) drug resistance prevalence was 27.4% (95% confidence interval (CI): 22.8%-32.6%). However, the total resistance was about 10%, when calculated among all HIV-positive survey participants (virologically suppressed and non-suppressed), similar to our model-based estimates (median: 15%; IQR: 7.5%-25.3%). Survey-based drug resistance prevalence was 55.7% among participants with positive ARV detection, of which 14.3% was NNRTI-only and 40.4% was NNRTI+NRTI resistance. Among those who tested negative for ARVs, resistance prevalence was 22.8%, comprised by 20% NNRTI-only and 2.1% NNRTI+NRTI resistance; rising to 75.9% among ARV-experienced, while falling to 15.3% among ARV-naïve participants. Although differences in study design prevent a direct comparison, these survey data are similar to our model-based resistance estimates (10% vs 15% total, 42.6%-67.9% (ARV+) vs 80% (ART-adjusted) total and 29.6%-52.2% (ARV+) vs 55% (ART-adjusted) NNRTI+NRTI acquired resistance). Of note, i) our modeling context is KwaZulu-Natal, the South African province with the highest burden of HIV, ii) the denominator for our ART-adjusted estimates includes all HIV-positive individuals with ART-experience and virological non-suppression, and iii) our model allows for re-entry into ART; these factors may explain the somewhat lower survey-based compared to model-based estimates of drug resistance prevalence overall, as well as NRTI-resistant (versus NNRTI-resistant) majority viruses given their dynamics (Table 1).

## Discussion

As the world gears up to end the HIV/AIDS epidemic as a public health threat by 2030, primarily through universal ART, this study addresses the spread of HIV drug resistance from failure of the preferred first-line antiretroviral regimens containing TDF + XTC + NNRTI. Using a novel, detailed and well-parametrized individual-based model of the HIV epidemic in KwaZulu-Natal, we project the prevalence and incidence of HIV drug resistance from ART, in the majority virus at the population level over time, through simulations of CD4-based or Fast-track approaches of ART implementation. The following are important insights from our study. 1) The total resistance prevalence (proportion of HIV-infected individuals with virological non-suppression and acquired and/or transmitted majority drug resistance) from treatment increases over time, especially with the CD4-based approach. 2) By 2030, total resistance from the implementation of Fast-track, is less compared to that from CD4-based ART. 3) Acquired resistance to first-line ART predominantly constitutes the total drug resistance prevalence. 4) Most individuals with acquired drug resistance harbor dual or triple major drug mutations. 5) The proportion of incident/new infections with transmitted drug-resistant HIV increases over time. 6) NNRTI-associated mutations in the majority virus are predominant among the prevalent, transmitted drug-resistant HIV infections. 7) Though differences in study design preclude an exact comparison, our model-based projections of moderate-to-high levels of drug resistance at 2018, are in general agreement with survey- and surveillance-based data [14, 17, 81].

Our base case and uncertainty analyses project increasing levels of total resistance prevalence in the scenario of CD4-based ART reaching a mean (median) value of 30% (31.4%) by 2030 compared to 15% at 2018. The total resistance appears to decrease to 13% (14.5%) in the Fast-track scenario by 2030. Elsewhere, we have found that the principal drivers for decrease in resistance with Fast-track ART are increasingly effective adherence support and rapid initiation of second-line after failure of first-line ART.

We predict that most individuals with acquired resistance have dual or triple drug mutations. In addition, the prevalence estimates of signature mutations associated with TDF and XTC and the NNRTI class mutations, are similarly high (~80%). These results underscore the importance of timely and equitable utilization of second-line ART. Though first-line ART associated mutations were not found to compromise the efficacy of second-line ART in clinical trials [72, 82, 83], our findings warrant caution, particularly over long term.

Our modeling shows that the proportion of incident infections with transmitted drug-resistant HIV increases over time with the implementation of ART. At 2018, our predicted value is 18%, which exceeds the WHO’s 10% threshold for pretreatment drug resistance (detected in antiretroviral naïve or antiretroviral exposed individuals initiating or reinitiating first-line ART) [6], and climbs to 40% by 2030. These data highlight the criticality of universal access to alternate first-line ART regimens and/or point of care drug resistance testing. We do not report the trade-off between averted infections and transmitted drug resistance due to ART, nor the epidemiological or economic impact of drug resistance; as these issues have been examined previously by us [31, 33, 84] and others [67, 85, 86]. Averted infections may offset the risk of resistance over short term [67]. By contrast, in contexts where current levels of pretreatment drug resistance exceed 10%, it is estimated that 16% (890, 000) of AIDS deaths, 9% (450, 000) of new infections, and 8% ($6.5 billion) of ART program costs in sub-Saharan Africa in 2016–2030 will be attributable to HIV drug resistance [85].

Similar to other modeling studies this work has limitations. Precise details of our model's projections will be affected by variations in the embedded structural and parameter assumptions, especially those regarding sexual behavior. Nevertheless, we used rigorous model construction, calibration, parameterization, and analyses. Our assumptions regarding drug resistance were derived from disparate literature sources; however, we carefully parameterized our model inputs, incorporating relevant current and local data, and explored plausible input ranges in uncertainty analyses. Our study focuses on drug resistance from the scale-up of WHO’s preferred first-line ART regimens containing TDF + XTC + EFV/NVP. We do not model the recommended consideration of non-NNRTI (dolutegravir-containing) first-line ART for all starters in countries with high (≥ 10%) pretreatment drug resistance to EFV/NVP [6, 87] or the interim guidelines that recommend changing the preferred first- and second-line regimens as dolutegravir-based [5]. Data from South Africa [8–17] are mixed regarding drug resistance at the population-level, though a study suggests that KwaZulu-Natal may have surpassed the 10% pretreatment resistance threshold [17]. Though dolutegravir rollout in South Africa appears imminent, the incorporation of this scenario in our model is challenging for the following reasons. The timing, pace and scale of dolutegravir roll out in South Africa are not precisely known [79, 80]. The policy of dolutegravir implementation may change from use in people initiating ART to use in all people on ART [88]. Several gaps in the evidence base need to be addressed by researchers as part of dolutegravir roll out [89]: More data are required to determine the risk of adverse birth outcomes when women initiate dolutegravir-based regimens before conception; While increasing access to viral load testing for monitoring the effectiveness of dolutegravir remains crucial, the best strategy to manage patients with viremia is unclear; The evidence to support the effectiveness of dolutegravir when given with tuberculosis treatment remains scarce. Finally, whether NRTI resistance will affect the long-term efficacy of dolutegravir-based regimens in first-line, and potentially second-line, ART is unknown [90] and dolutegravir-resistance patterns may differ across HIV type 1 non-B subtypes [91]. Clinical trials, cohorts, and surveillance of HIV drug resistance will be necessary to answer these questions, maximize the benefits of dolutegravir-based regimens, as well as inform future mathematical modeling. Nevertheless, our study provides critical insight into the potential trends and patterns of HIV drug resistance in South Africa, in the context of scale-up of TDF + XTC + EFV/NVP, as the preferred first-line ART, at present and in the future; lending support to the WHO recommendations for regimen change [5]. Future work will address model refinements informed by new available data. We do not include the prevention of mother to child transmission, which may be an important source of resistance [92]. Finally, our modeling context is the mature, generalized, high-prevalence HIV epidemic in KwaZulu-Natal, South Africa. While our quantitative findings may not be directly generalizable to other contexts, the qualitative insights from our modeling are robust.

## Conclusion

Monitoring, prevention and treatment of drug-resistant HIV are vital components of the HIV response. Affordable and universal access to safe and effective first- and subsequent-line ART regimens, alongside reliable and convenient tests for HIV viral load and drug resistance, are crucial for the end of AIDS.

## Supporting information

S1 Table Behavioral, epidemiological and demographic model input parameters. (DOCX)

S1 Fig Survey-based data. Human Sciences Research Council survey-based HIV drug resistance in South Africa. The illustration was generated from publically available data.
(TIF)
