# Supplementary material for: Drug resistance from preferred antiretroviral regimens for HIV infection in South Africa: A modeling study
Source: PLoS One. 2019 Jul 3;14(7):e0218649. doi: 10.1371/journal.pone.0218649 (PMC6609148; doi:10.1371/journal.pone.0218649)
Supplement: S1 Table — (DOCX) [file pone.0218649.s001.docx]

| Parameter | Value | Source |
| --- | --- | --- |
| **Sexual behavior parameters** |  |  |
|  |  |  |
| Average partner change rate, per year | 1.15 | [1] |
| Relative partner change rate by age (women, men) |  | [2, 3] |
| 15–19 | 0.81, 0.10 |  |
| 20–24 | 1.41, 0.76 |  |
| 25–29 | 1.55, 1.87 |  |
| 30–34 | 1.24, 2.18 |  |
| 35–39 | 0.80, 1.49 |  |
| 40–44 | 0.43, 0.69 |  |
| 45–49 | 0.21, 0.23 |  |
| 50–54 | 0.09, 0.06 |  |
| Relative partner change rate by sexual activity level (women, men) |  | [4, 5] |
| Least | 0.33, 0.01 |  |
| Low | 0.63, 0.32 |  |
| Medium | 1.45, 3.88 |  |
| High | 114.23, 13.54 |  |
| Proportion of individuals initially in each sexual activity level (women, men), % |  |  |
| Least | 10.4, 8.0 | [1] |
| Low | 38.6, 35.5 | Calculated |
| Medium | 0.8, 5.2 | [1, 6) |
| High | 0.2, 1.4 | [7, 8] |
| Average duration of female sex work, years | 2.7 | [9) |
| Assortativity of sexual mixing by sexual activity level | 0.43 | [2] |
| Assortativity of sexual mixing between men and women of the same age | 0.30 | [2) |
| Assortativity of sexual mixing between younger women and men five years older | 0.76 | [2] |
| Number of sexual acts per regular partnership* | 115 | [10-12) |
| Number of sexual acts per casual partnership* | 12.4 | [4] |
| Number of sexual acts per short partnership* | 3.3 | [4) |
| Condom use |  |  |
| Effectiveness of condoms for HIV prevention, % | 90 | [13] |
| Year that condom use starts to increase | 1999.3 | [14) |
| Year that condom use stabilizes | 2006.5 | [14] |
| Condom use frequency in regular partnerships* (initially, after increase), % | 2, 46 | [1, 14] |
| Condom use frequency in casual partnerships* (initially, after increase), % | 13, 52 | [1, 14] |
| Condom use frequency in short partnerships* (initially, after increase), % | 34, 64 | [1, 14] |
|  |  |  |
| **Epidemiological parameters** |  |  |
| Epidemic start date | 1982 | Assumed |
| HIV transmission probability, % per sexual act |  |  |
| Acute infection | 1.395 | [15-19] |
| Chronic infection | 0.247 | [15-18, 20] |
| AIDS | 0.489 | [15-18, 20] |
| HIV disease progression |  |  |
| Duration of acute infection pre-seroconversion, weeks | 3.01 | [21, 22] |
| Duration of acute infection post-seroconversion, weeks | 9.50 | [21, 22] |
| Time from onset of chronic infection until CD4 ≤ 500 cells/µL, years | 1.01 | [23] |
| Time from CD4 ≤ 500 cells/µL until CD4 ≤ 350 cells/µL, years | 3.00 | [23] |
| Time from CD4 ≤ 350 cells/µL until CD4 ≤ 200 cells/µL, years | 3.75 | [23] |
| Time from CD4 ≤ 200 cells/µL until death, years | 3.32 | [24] |
|  |  |  |
| **Demographic parameters** |  |  |
| Initial population size (simulation begins at 1978) | 2,466,000 |  |
| Initial population age distribution, % |  | [25] |
| 15–19 | 19 |  |
| 20–24 | 18 |  |
| 25–29 | 16 |  |
| 30–34 | 13 |  |
| 35–39 | 11 |  |
| 40–44 | 9 |  |
| 45–49 | 8 |  |
| 50–54 | 6 |  |
| HIV-unrelated mortality rates (women, men), per year |  | [26] |
| 15–19 | 0.00076, 0.00179 |  |
| 20–24 | 0.00129, 0.00370 |  |
| 25–29 | 0.00182, 0.00480 |  |
| 30–34 | 0.00250, 0.00574 |  |
| 35–39 | 0.00313, 0.00687 |  |
| 40–44 | 0.00444, 0.00930 |  |
| 45–49 | 0.00572, 0.01161 |  |
| 50–54 | 0.00958, 0.01690 |  |
| 55+ | 0.04694, 0.05828 |  |

Behavioral and epidemiological parameter values were calibrated via Bayesian melding as described in detail elsewhere [27, 28]. Prior distributions on these inputs were informed by the sources listed.

* Partnerships are categorized as “regular” if at least one partner is in the least or low sexual activity level, “short” if both partners are in the high sexual activity level, and “casual” otherwise.

**References**

1. McGrath N, Eaton JW, Bärnighausen TW, Tanser F, Newell M-L. Sexual behaviour in a rural high HIV prevalence South African community: time trends in the antiretroviral treatment era. AIDS. 2013;27(15):2461-70.

2. Garnett GP, Anderson RM. Factors controlling the spread of HIV in heterosexual communities in developing countries: patterns of mixing between different age and sexual activity classes. Philos Trans R Soc Lond B Biol Sci. 1993;342(1300):137-59.

3. Johnson L, Dorrington R, Bradshaw D, Pillay-Van Wyk V, Rehle T. Sexual behaviour patterns in South Africa and their association with the spread of HIV: insights from a mathematical model. Demographic Research. 2009;21(11):289-340.

4. South African Centre for Epidemiological Modelling and Analysis. The Modes of Transmission of HIV in South Africa. Stellenbosch: South African Centre for Epidemiological Modelling and Analysis; 2009.

5. Ramjee G, Weber AE, Morar NS. Recording sexual behavior: comparison of recall questionnaires with a coital diary. Sexually Transmitted Diseases. 1999;26(7):374-80.

6. Shisana O, Rehle T, Simbayi L, Zuma K, Jooste S, Zungu N, et al. South African National HIV Prevalence, Incidence and Behaviour Survey, 2012. Cape Town: HSRC Press; 2014.

7. Caraël M, Slaymaker E, Lyerla R, Sarkar S. Clients of sex workers in different regions of the world: hard to count. Sexually Transmitted Infections. 2006;82(Suppl 3):iii26-iii33.

8. Vandepitte J, Lyerla R, Dallabetta G, Crabbé F, Alary M, Buvé A. Estimates of the number of female sex workers in different regions of the world. Sexually Transmitted Infections. 2006;82(Suppl 3):iii18-iii25.

9. Scorgie F, Chersich M, Ntaganira I, Gerbase A, Lule F, Lo Y-R. Socio-demographic characteristics and behavioral risk factors of female sex workers in sub-Saharan Africa: a systematic review. AIDS and Behavior. 2012;16(4):920-33.

10. Gray RH, Wawer MJ, Brookmeyer R, Sewankambo NK, Serwadda D, Wabwire-Mangen F, et al. Probability of HIV-1 transmission per coital act in monogamous, heterosexual, HIV-1-discordant couples in Rakai, Uganda. The Lancet. 2001;357(9263):1149-53.

11. Brown MS. Coitus, the proximate determinant of conception: inter-country variance in sub-Saharan Africa. Journal of Biosocial Science. 2000;32(2):145-59.

12. Myer L, Mathews C, Little F. Condom use and sexual behaviors among individuals procuring free male condoms in South Africa: a prospective study. Sexually Transmitted Diseases. 2002;29(4):239-41.

13. Weller SC, Davis-Beaty K. Condom effectiveness in reducing heterosexual HIV transmission. Cochrane Database of Systematic Reviews. 2002(1):CD003255.

14. Cremin I, Alsallaq R, Dybul M, Piot P, Garnett G, Hallett TB. The new role of antiretrovirals in combination HIV prevention: a mathematical modelling analysis. AIDS. 2013;27(3):447-58.

15. Boily M-C, Baggaley RF, Wang L, Mâsse B, White RG, Hayes RJ, et al. Heterosexual risk of HIV-1 infection per sexual act: systematic review and meta-analysis of observational studies. The Lancet Infectious Diseases. 2009;9(2):118-29.

16. Quinn TC, Wawer MJ, Sewankambo N, Serwadda D, Li C, Wabwire-Mangen F, et al. Viral load and heterosexual transmission of human immunodeficiency virus type 1. New England Journal of Medicine. 2000;342(13):921-9.

17. Wawer MJ, Gray RH, Sewankambo NK, Serwadda D, Li X, Laeyendecker O, et al. Rates of HIV-1 transmission per coital act, by stage of HIV-1 infection, in Rakai, Uganda. J Infect Dis. 2005;191(9):1403-9.

18. Hughes JP, Baeten JM, Lingappa JR, Magaret AS, Wald A, de Bruyn G, et al. Determinants of per-coital-act HIV-1 infectivity among African HIV-1-serodiscordant couples. The Journal of Infectious Diseases. 2012;205(3):358-65.

19. Pilcher CD, Price MA, Hoffman IF, Galvin S, Martinson FEA, Kazembe PN, et al. Frequent detection of acute primary HIV infection in men in Malawi. AIDS. 2004;18(3):517-24.

20. Novitsky V, Wang R, Bussmann H, Lockman S, Baum M, Shapiro R, et al. HIV-1 subtype C-infected individuals maintaining high viral load as potential targets for the "test-and-treat" approach to reduce HIV transmission. PLoS One. 2010;5(4):e10148.

21. Cohen MS, Gay CL, Busch MP, Hecht FM. The detection of acute HIV infection. The Journal of Infectious Diseases. 2010;202(Suppl 2):S270-S7.

22. Fiebig EW, Wright DJ, Rawal BD, Garrett PE, Schumacher RT, Peddada L, et al. Dynamics of HIV viremia and antibody seroconversion in plasma donors: implications for diagnosis and staging of primary HIV infection. AIDS. 2003;17(13):1871-9.

23. Lodi S, Phillips A, Touloumi G, Geskus R, Meyer L, Thiébaut R, et al. Time from human immunodeficiency virus seroconversion to reaching CD4+ cell count thresholds <200, <350, and <500 cells/mm^3^: assessment of need following changes in treatment guidelines. Clinical Infectious Diseases. 2011;53(8):817-25.

24. Van der Paal L, Shafer LA, Todd J, Mayanja BN, Whitworth JAG, Grosskurth H. HIV-1 disease progression and mortality before the introduction of highly active antiretroviral therapy in rural Uganda. AIDS. 2007;21(Suppl 6):S21-S9.

25. U.S. Census Bureau. International data base: South Africa, 1985 Washington D.C.: U.S. Census Bureau; 2014 [Available from: <http://www.census.gov/population/international/data/idb/informationGateway.php>.

26. World Health Organization. Global Health Observatory Data Repository World Health Organization, Geneva2013 [South Africa life tables]. Available from: <http://apps.who.int/gho/data/node.main>.

27. Glaubius RL, Hood G, Penrose KJ, Parikh UM, Mellors JW, Bendavid E, et al. Cost-effectiveness of Injectable Preexposure Prophylaxis for HIV Prevention in South Africa. Clin Infect Dis. 2016;63(4):539-47.

28. Glaubius RL, Parikh UM, Hood G, Penrose KJ, Bendavid E, Mellors JW, et al. Deciphering the Effects of Injectable Pre-Exposure Prophylaxis for Combination HIV Prevention. Open Forum Infectious Diseases. 2016.
