# Supplementary figures and images for: Drug resistance from preferred antiretroviral regimens for HIV infection in South Africa: A modeling study
Source: PLoS One. 2019 Jul 3;14(7):e0218649. doi: 10.1371/journal.pone.0218649 (PMC6609148; doi:10.1371/journal.pone.0218649)

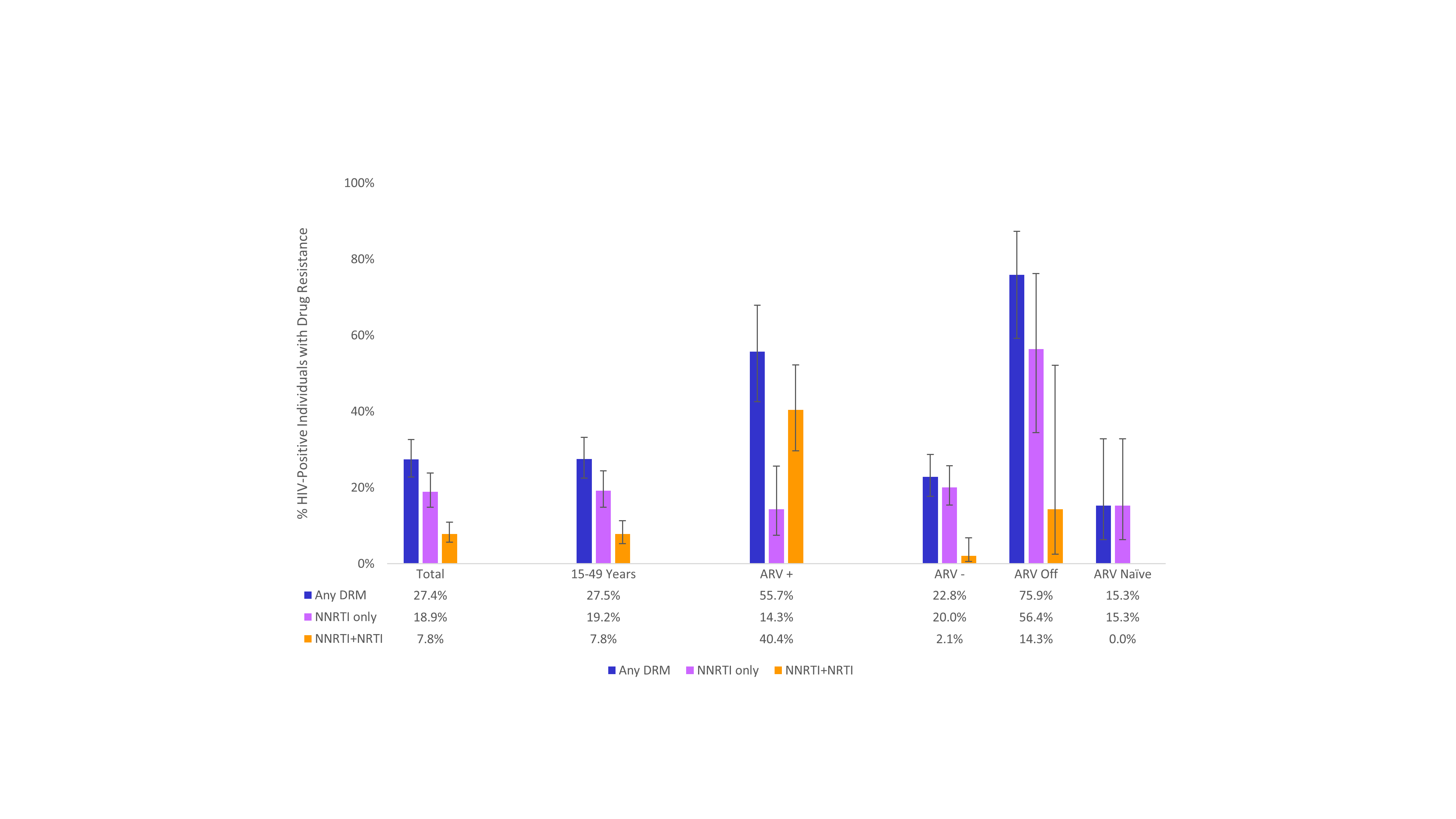

Supplement: S1 Fig — Human Sciences Research Council survey-based HIV drug resistance in South Africa. The illustration was generated from publically available data. (TIF) [file pone.0218649.s002.tif]
